# Fruit Fiber Consumption Specifically Improves Liver Health Status in Obese Subjects under Energy Restriction

**DOI:** 10.3390/nu9070667

**Published:** 2017-06-28

**Authors:** Irene Cantero, Itziar Abete, J. Ignacio Monreal, J. Alfredo Martinez, M. Angeles Zulet

**Affiliations:** 1 Department of Nutrition, Food Science and Physiology, Faculty of Pharmacy and Nutrition, University of Navarra, 31008 Pamplona, Spain; icantero.1@alumni.unav.es (I.C.); iabetego@unav.es (I.A.); mazulet@unav.es (M.A.Z.); 2 Centre for Nutrition Research, Faculty of Pharmacy and Nutrition, University of Navarra, 31008 Pamplona, Spain; 3 CIBERObn, Physiopathology of obesity and nutrition, Instituto de Salud Carlos III, 28029 Madrid, Spain; 4 Navarra Institute for Health Research (IdiSNA), 31008 Pamplona, Spain; jimonreal@unav.es or jimonreal@unav.es; 5 Clinical Chemistry Department, University Clinic of Navarra, University of Navarra, 31008 Pamplona, Spain

**Keywords:** obesity, fatty liver disease, metabolic syndrome, insoluble fiber, fiber, AHA, RESMENA

## Abstract

The prevalence of non-alcoholic-fatty-liver-disease (NAFLD) is associated with obesity, diabetes, and metabolic syndrome (MS). This study aimed to evaluate the influence of two energy-restricted diets on non-invasive markers and scores of liver damage in obese individuals with features of MS after six months of follow-up and to assess the role of fiber content in metabolic outcomes. Seventy obese individuals from the RESMENA (Reduction of Metabolic Syndrome in Navarra) study were evaluated at baseline and after six months of energy-restricted nutritional intervention (American Heart Association (AHA) and RESMENA dietary groups). Dietary records, anthropometrical data, body composition by dual energy X-ray absorptiometry (DXA), and routine laboratory measurements were analyzed by standardized methods. Regarding liver status, cytokeratin-18 fragments and several non-invasive scores of fatty liver were also assessed. The RESMENA strategy was a good and complementary alternative to AHA for the treatment of obesity-related comorbidities. Participants with higher insoluble fiber consumption (≥7.5 g/day) showed improvements in fatty liver index (FLI), hepatic steatosis index (HIS), and NAFLD liver fat score (NAFLD_LFS), while gamma-glutamyl transferase (GGT) and transaminases evidenced significant improvements as a result of fruit fiber consumption (≥8.8 g/day). Remarkably, a regression model evidenced a relationship between liver status and fiber from fruits. These results support the design of dietary patterns based on the consumption of insoluble fiber and fiber from fruits in the context of energy restriction for the management of obese patients suffering fatty liver disease.

## 1. Introduction

Non-alcoholic fatty liver disease (NAFLD) is a condition of hepatic steatosis in the absence of excessive alcohol consumption [1]. The spectrum of NAFLD ranges from simple steatosis to non-alcoholic steatohepatitis (NASH), which can lead to fibrosis and finally hepatocellular carcinoma [2]. As a consequence of the obesity epidemic, this liver pathology (NAFLD) is emerging as an important public health issue and is the most common cause of chronic liver disease in Western countries, with a prevalence of about 20–30% the general adult population [1]. The pathogenesis of NAFLD is multifactorial and triggered by environmental factors such as unbalanced diets or/and overnutrition as well as by sedentary lifestyles [1]. Concerning the diagnosis of NAFLD, liver biopsy is considered the “gold standard” of steatosis, fibrosis, and cirrhosis. However, it is rarely performed because it is an invasive procedure with a significant degree of sampling error [2]. Thus, investigators are focusing on the design and application of non-invasive liver damage scores for the diagnosis and management of liver disease [3].

Nowadays, the treatment of NAFLD is based on lifestyle modifications, such as changes in dietary patterns [4]. Thus, weight loss, exercise, and healthy eating habits are the main strategies to reduce the incidence and prevalence of NAFLD, although the metabolic mechanisms are still poorly understood [5]. Previous studies have evidenced a role of weight loss on NAFLD management [6]. However, further investigations to elucidate the interplay between specific dietary components and fatty liver combined with weight loss are necessary [7], as the number of randomized controlled studies conducted in this area remain scarce [8]. In this context, the aim of this study was to evaluate the influence of two energy-restricted diets on non-invasive markers and scores of liver damage in obese individuals with features of metabolic syndrome after six months of follow-up and to assess the role of fiber content (quality and quantity) in metabolic outcomes.

## 2. Materials and Methods 

### 2.1. Study Design

**Figure 1:**
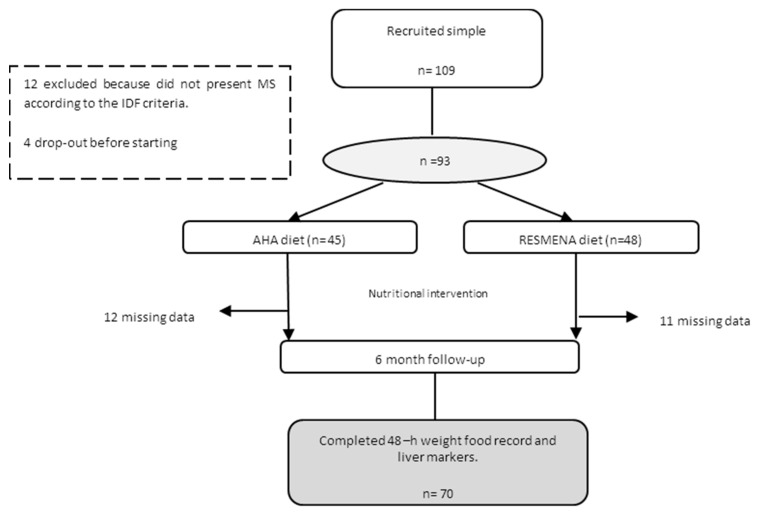
Study design flowchart. AHA: American Heart Association; IDF: International Diabetes Federation; MS: metabolic syndrome.

The current study included a total of 70 subjects from the RESMENA (Reduction of Metabolic Syndrome in Navarra) study, which was designed as a randomized controlled intervention trial to compare the effects of two hypocaloric dietary strategies on metabolic syndrome (MS) comorbidities after six-months of follow-up [9]. Initially, a total of 109 Caucasian adults were enrolled in the study, however, 12 did not present MS according to the International Diabetes Federation (IDF) criteria when the study began, and another four subjects decided not to start the dietary treatment after signing the written informed consent. Therefore, 93 subjects were assigned using the “random between 1 and 2” function in the Microsoft Office Excel 2003 software (Microsoft Iberica, Barcelona, Spain) to follow one of the two energy-restricted diets. After six months of weight loss intervention, twenty-three participants (11 from RESMENA and 12 from the American Heart Association (AHA) dietary group) did not complete the dietary intake questionnaire and/or were missing data regarding biomarkers of liver status at the end of the intervention. Thus, a total of 70 participants had complete information to carry out the objective of this study (Figure 1). Subjects were asked to maintain their usual physical activity (MET—metabolic equivalent of the task), which was controlled by a 24-h physical activity questionnaire administered at the beginning and at the end of the study.

The RESMENA study followed the CONSORT 2010 guidelines, except for blinding. This research was performed according to the ethical guidelines of the Declaration of Helsinki, and was appropriately registered (www.clinicaltrials.gov; NCT01087086). The study was approved by the Research Ethics Committee of the University of Navarra (ref. 065/2009). Additional aspects of this intervention trial have been detailed elsewhere [9].

### 2.2. Nutritional Intervention 

Two hypocaloric dietary patterns (AHA vs. RESMENA)—both with the same energy restriction (−30% of the individual’s requirements)—were prescribed and compared. The AHA diet was based on American Heart Association guidelines, including 3–5 meals/day, a macronutrient distribution of 55% total caloric value (TCV) from carbohydrates (whole grains were recommended, but not mandatory), 15% from proteins, and 30% from lipids. On the other hand, the RESMENA diet was designed with a higher meal frequency, consisting of seven meals/day, and a macronutrient distribution of 40% TCV from carbohydrates (whole grains were required), 30% from proteins (mainly vegetable protein), and 30% from lipids (omega-3 and extra virgin olive oil intake required).

A 48-h weighed food record was collected at the beginning and at the end of the study, and was used to assess the volunteer’s adherence to the prescribed diet. The energy and nutrient content of these questionnaires were determined using the DIAL software (Alce Ingenieria, Madrid, Spain), as described elsewhere [10]. This is a validated program in Spain, designed with Spanish foods, and provides information regarding grams of insoluble and soluble fiber obtained from the diet, as well as the total fiber supplied by different food groups (fiber from fruits, vegetables, or cereals, separately). 

Anthropometric measurements were assessed in fasting conditions following standardized procedures, as previously reported [9]. Body weight, waist circumference (WC), and body composition as assessed by dual-energy X-ray absorptiometry (Lunar Prodigy, software version 6.0, Madison, WI, USA) were examined at baseline and at the end of the intervention using validated protocols [10]. Body mass index (BMI) was calculated as body weight divided by squared height (kg/m^2^). Conicity index (CI) was calculated as the (waist circumference/(0.109 × square root of weight/height)) as published [11], where WC and height were measured in meters and weight was measured in kg. Blood glucose, total cholesterol (TC), triglycerides (TG), alanine aminotransferase (ALT), aspartate aminotransferase (AST), and gamma-glutamyl transferase (GGT) were measured on an autoanalyzer (Pentra C-200; HORIBA ABX, Madrid, Spain). Plasma concentrations of cytokeratin-18 (CK18)-fragments (M30 and M65) levels were assessed by ELISA assay (Mercodia, Uppsala, Sweden) with an autoanalyzer system (Triturus, Grifols SA, Barcelona, Spain) following the manufacturer’s instructions.

The fatty liver index (FLI) is an algorithm derived from serum TG, BMI, WC, and GGT levels [12,13,14,15], and was validated in a large group of subjects with or without suspected liver disease with an accuracy of 0.84 (95% CI) in detecting fatty liver. Fatty liver index varies between 0 and 100, indicating the presence of steatosis with a score ≥ 60. The NAFLD liver fat score [16] was calculated by a complex equation, combining the following parameters: the presence of MS (according to IDF criteria), the presence of Type 2 diabetes, fasting serum insulin, aspartate amino-transferase (AST), and the aspartate–alanine aminotransferase ratio (AST/ALT ratio). This score (NAFLD_LFS) allows the prediction of steatosis, defined as a liver fat content ≥ 5.56% as assessed by ^1^H-magnetic resonance spectroscopy (^1^H-MRS) with good accuracy (area under the receiver operating characteristic curve (AUROC): 0.86). The hepatic steatosis index (HSI) was also calculated for the assessment of liver steatosis. The formula includes ALT/AST ratio, diabetes, and gender [17]. A value > 36 indicates liver steatosis. Additional hepatic steatosis predictors were calculated based on the available data such as the BAAT (body mass index, age, alanine aminotransferase, triglycerides) and BARD (body mass index, aspartate aminotransferase:alanine aminotransferase, diabetes) scores which included BMI, age, alanine amino transferase (ALT), and TG; and BMI, AST/ALT ratio and presence of diabetes, respectively [18]. Finally, the visceral adiposity index (VAI) was calculated [19], given that is a simple index of visceral fat function that predicts cardiometabolic risk in general population. 

### 2.3. Statistical Analysis 

Analyses were performed using 12.0 (Stata Corp College Station, TX, USA). Fiber dietary groups were classified as total fiber consumption (total dietary fiber), insoluble fiber (total insoluble dietary fiber), soluble fiber (total soluble dietary fiber), fruit fiber (specifically fiber from fruits), vegetable fiber (specifically fiber from vegetables), and cereal fiber (specifically fiber from cereals). The median values of fiber intake were used to classify the participants into high (≥50th percentile) or low (<50th percentile) fiber consumption. Normality distributions of the evaluated variables were determined by Shapiro–Wilk test. Continuous variables were compared between groups by the Student’s *t*-test or the Mann–Whitney *U* test for parametric or non-parametric variables, respectively. Categorical variables were compared by the chi-squared test. The relationship between variables was assessed by the Pearson’s correlation coefficient or the Spearman’s rho (*p*). A linear regression model was performed to assess the influence of independent variables such as fiber from fruits, age, total energy intake, and physical activity estimations on the variability of FLI score. The linear regression model was not adjusted for weight variable, since FLI carries the BMI value in its calculation. All *p*-values presented are two-tailed, and differences were considered statistically significant at *p* < 0.05.

## 3. Results

The average age of participants was 49 ± 9 years, of which 50.5% were women. At the beginning of the study, no significant differences were observed between dietary groups. After 6 months, the mean of body weight loss was 7.9 ± 4.8 kg and 9.7 ± 5.4 kg in the AHA and RESMENA dietary groups, respectively (Table 1). Both nutritional treatments significantly reduced BMI, CI, WC, total and android fat mass, as well as IDF (International Diabetes Federation) of metabolic syndrome after 6 months of nutritional intervention. Likewise, the cardiometabolic risk factors (Triglycerides/glucose ratio (TyG index), waist-to-height ratio, % diabetes, % hypertension) showed significant reductions with both diets. However, the changes (baseline vs. 6 months) in the reported variables did not differ between the RESMENA and AHA dietary strategies, with the exception of high-density lipoprotein cholesterol (HDL-c) levels, which were significantly increased in AHA compared to the RESMENA group at the end of study (Table 1). Regarding dietary intake, the analysis of weighed dietary records showed that total fiber intake was higher during the intervention period than the intake before starting the nutritional program (baseline) in both dietary groups, while participants from the RESMENA dietary group showed a significantly higher intake of insoluble fiber after six months (Table 1).

In relation to liver health, a significant decrease was observed in ALT, GGT, and M30 levels as well as NAFLD_LF score, FLI, HSI, VAI and BAAT scores with both dietary strategies (AHA and RESMENA) after 6 months of follow-up (Table 2). Given that transaminases showed significant difference at baseline between dietary groups, the analyses concerning transaminases were adjusted by ALT and AST values at baseline. AST values only showed a significant reduction in the RESMENA group. In contrast, fragments of M65 only obtained significant improvements in the AHA group. In addition, the BARD score did not show relevant improvements either dietary group. It is important to note that the changes (baseline vs. 6 months) in the reported variables were not different between dietary strategies (Table 2). Thus, both dietary strategies were equally effective regarding liver status. Consequently and based on previous investigations [20,21], both dietary groups were merged for the analysis of fiber consumption because they produced similar outcomes for all relevant variables and markers.

We analyzed the potential role of dietary macronutrients (carbohydrates, fat, and proteins) on liver status (FLI), and no significant associations were observed: carbohydrates (β = 0.011; *p* = 0.751; *R* = 0.0017), lipids (β = 0.126; *p* = 0.084; *R* = 0.049), saturated fatty acids (β = −0.017; *p* = 0.431, *R* = 0.009), monounsaturated fatty acids (β = −0.031, *p* =0.390, *R* = 0.001), polyunsaturated fatty acids (β = −0.198; *p* = 0.209; *R* = 0.208), omega-3 fatty acids (β = −0.930; *p* = 0.288; *R* = 0.051), and proteins β = −0.66; *p* = 0.480; *R* = 0.008). In order to analyze the specific role of fiber on liver health, the predictors of liver damage according to high and low (<50th percentile vs. ≥50th percentile) consumption of different types of fiber were assessed at the end of the study (Table 3). Insoluble fiber and fiber from fruits showed interesting results, since participants with higher consumption of these types of fiber reduced markers of liver status associated with fatty liver. It is important to highlight that a high consumption of insoluble fiber resulted in improvements in FLI, HSI, NAFLD_LFS, while significant improvements in GGT and transaminases (AST and ALT) were observed as a result of fruit fiber consumption.

**Figure 2:**
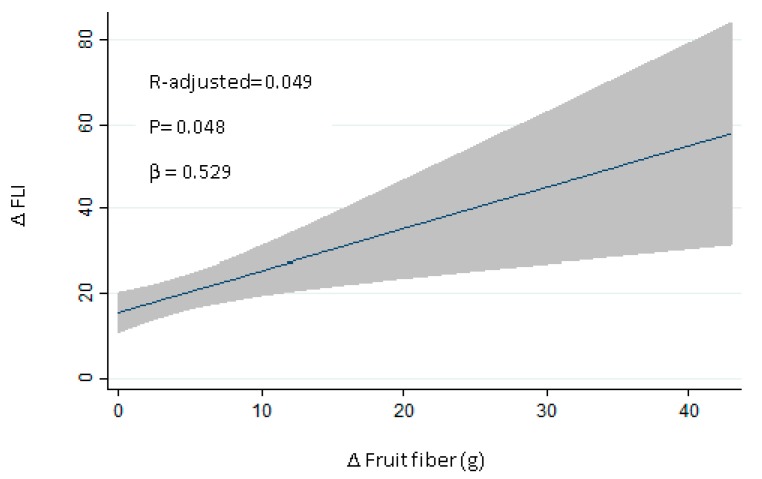
Regression analysis with changes in FLI and fruit fiber.

Reinforcing this idea, a linear regression analysis was carried out to assess the influence of changes in the consumption of different types of fiber in changes in the FLI. Fiber from fruits demonstrated a positive relationship (*R*-adjusted: 0.049; *p* = 0.048; Figure 2). The effect of each fiber group was also analyzed: vegetables (β = 0.287; *p* = 0.138, *R* = 0.037), cereals (β = −0.295; *p* = 0.608, *R* = 0.004), insoluble (β = 0.491; *p* = 0.159, *R* = 0.033), soluble (β = −0.472; *p* = 0.545, *R* = 0.002). The linear regression model was adjusted by age, total energy (kcal), and physical activity (metabolic equivalent of the task, MET) changes (Table 4). When these variables were jointly considered, the predictors of the model explained up to 11.6% of the variation of changes in the FLI (adjusted *R*^2^ = 0.116; P_model_ < 0.026). 

## 4. Discussion

The design of new dietary strategies for NAFLD prevention and for the reduction of causal factors is needed [22]. Increasing rates of obesity and MS are having an important impact on the rising incidence and prevalence of liver diseases such as NAFLD; thus, well-designed dietary interventions are necessary [22]. In this context, the present study compared the effects of two energy-restricted dietary strategies on anthropometric, biochemical, and non-invasive markers of liver status in obese individuals with MS features after 6 months of follow-up. Regarding weight loss, body composition, IDF criteria, and cardiometabolic risk, both dietary regimens had equal effect on most metabolic markers. HDL-c concentrations were increased in both dietary groups, but only reached significance in the AHA group. This outcome might be expected, since the AHA diet is specifically based on the AHA guidelines [23], which focus on cardiovascular care and therefore on lipid profile management. Interestingly, the RESMENA diet had a positive effect on body weight by reducing android fat mass—a body region which has been commonly associated with hepatic steatosis [24]. On the other hand, current research has focused on identifying biomarkers to predict NASH or NAFLD [25,26]. In this context, Bedogni et al. (2006) designed a simple scoring system named FLI which includes TG, GGT, BMI, and WC, and it is easily calculated [12]. FLI was developed for the prediction of fatty liver disease and shows a good area under the curve of 0.84 [26]. The accuracy of FLI in comparison with the ultrasonography method for detection and quantification of hepatic steatosis has been validated in several countries [12,27]. Furthermore, previous studies [17,28,29,30] have demonstrated and validated other non-invasive markers of liver status that were used in this study (CK18-fragments, HSI, VAI, BARD and BAAT score, NAFLD_LFS) in addition to GGT and transaminases. By employing these non-invasive biomarkers and scores to assess liver status, we showed that energy-restriction within both dietary patterns produced similar and significant beneficial effects on liver status, as shown in other dietary studies [31]. The BARD score did not indicate improvements after either of the nutritional interventions, which might be due to the fact that the BARD score is used to exclude advanced fibrosis in NAFLD, and other authors have noted that its sensitivity is low [30,32]. Considering the global results presented in this study, particularly improvements in android fat and liver status, we propose RESMENA as a novel dietary strategy to be explored in subjects with obesity and fatty liver. 

Energy restriction is a key factor for the management of NAFLD [33]. Indeed, the American Association for the Study of Liver Diseases (AASLD) recommends a loss of at least 3–5% of body weight to improve steatosis and a greater weight loss (up to 10%) to improve necroinflammation [34]. Other investigations have also demonstrated that weight loss interventions are effective in reducing NAFLD severity [6]. However, not only is the amount of energy a key component to be considered, but also the quality of the diet. In relation to fatty acids, diets rich in saturated fat and cholesterol and low in polyunsaturated fat have been associated with NASH [6], while omega-3 fatty acids have been proposed for the treatment of NAFLD or NASH [34,35]. In fact, the Mediterranean diet can reduce liver fat even in the absence of weight loss, and is the most recommended dietary pattern for NAFLD [36,37]. The Mediterranean diet is characterized by reduced carbohydrate intake (especially sugars and refined carbohydrates), and an increased intake of monounsaturated and omega-3 fatty acids. In the present study, both dietary patterns (AHA and RESMENA) included the same energy restriction (−30% E) and were designed taking into account the effect of a different distribution of macronutrients as well as the beneficial role of specific dietary components. In the present work, energy restriction was a key factor involved in the improvement of liver health; however, other dietary components—particularly fiber—may also be involved in the management of steatosis. Several studies have reported that fiber may play an important role in obesity and related diseases such as insulin resistance and liver diseases [38]. To our knowledge, this is the first study evaluating the association between types of fiber and liver status in subjects with obesity and metabolic syndrome following an energy-restricted diet. Although adherence to the dietary strategies was similar, resulting in no differences in macronutrient distribution, a variety of hepatic damage markers were lower in those individuals who had a higher fiber intake. In particular, fiber derived from fruit had a beneficial impact, suggesting that not only energy restriction but also other dietary components positively influence liver health. 

In this context, Zelber-Sagi et al., 2011 suggested that the consumption of diets higher in fiber could have a preventive role in hepatic disease [39]. Fiber contains components which are not classified as essential nutrients, but could be important mediators in human health. In fact, a pilot study of seven patients with NASH reported a significant decrease in aspartate aminotransferase levels compared to a placebo after 8 weeks of supplementation with oligofructose at 16 g/day [40]. Another study providing 10 g/day of psyllium fiber to 12 patients over the course of three months found a normalization of transaminases and GGT levels [41]. Dietary fiber can be divided in two groups—soluble and insoluble—based on physical, chemical, and functional characteristics [42]. Insoluble fibers are insoluble in water and gastric fluids. Soluble fiber dissolves in water and can resist gastrointestinal enzymatic digestion; therefore, soluble fiber can pass the small intestine to reach the colon where it can be fermented by intestinal microbiota [43]. This suggests that both soluble and insoluble fibers could have different roles in gastrointestinal health [44]. For this reason, different fiber types were investigated in the present study. Interestingly, we found two important fiber groups that could have a major effect on the reduction of predictors of liver heath: insoluble fiber and fiber from fruits. Although increasing dietary fiber has a favorable effect on body weight [45], and several studies suggest that fermentation activities of the gut microbiota may be a possible contributor to NAFLD [46], data regarding the effect of different types of fiber remain unclear [47]. Therefore, current research is warranted to determine the optimal dietary fibers for prevention and reduction of fatty liver disease accompanying obesity. 

A limitation of this study is that NAFLD was evaluated using non-invasive markers instead of imaging techniques and/or liver biopsies. However, the design of the current trial is based on validated non-invasive and affordable markers, which makes them an optimal form of diagnosis in clinical practice. In relation to improvements in liver status, other components occurring in the dietary pattern such us antioxidants, vitamins, fatty acids, energy restriction etc., may also explain the observed benefits on liver health, as they could work synergistically with fiber. In addition, comparisons involving means or regression analyses showed the same trend, although the statistical outcomes were more confirmatory when adjusted by age, total energy, and MET, which is in agreement with the expectations. The discordance between the results obtained with different analysis merits attention before concluding that all observed results are due to fruit fiber. On the other hand, one of the main strengths of the present research is that it is a randomized controlled trial, considered the gold standard in the hierarchy of research designs for evaluating the efficacy and safety of a nutritional intervention. Moreover, the fact that every dietary pattern has been personally designed for each patient, taking into account sex, height, initial body weight, and physical activity, should also be highlighted. Finally, it is important to point out that a well-recognized healthy dietary pattern (AHA) was used as reference, which demonstrates that the positive results obtained with the RESMENA diet are of significance.

## 5. Conclusions

These data support that the RESMENA diet may be a valid strategy to counteract MS features and liver damage accompanying obesity. In addition, this study reveals that the type of dietary fiber differentially impacts liver health status in obese subjects under energy restriction. However, these results should be interpreted with caution, since other components contained in fruits, in addition to fruit fiber, could be involved in the observed benefits.

## Figures and Tables

**Table 1:** Characteristics of the participants at baseline and after 6 months according to dietary treatment.

*n* = 70	AHA (*n* = 33)		RESMENA (*n* = 37)		Δ*p*-Value
	Baseline	6 Months	Baseline	6 Months	
**Body composition**					
Weight (kg)	99.2 (19)	91.8 (17) **	100.4 (16)	92.6 (16) **	0.155
BMI	35.8 (4)	32.9 (4) **	35.7 (4)	32.2 (4) **	0.153
Conicity Index	1.3	1.2 **	1.3	1.2 **	0.083
WC (cm)	110.2 (13)	103.3 (13) **	111.8 (12)	102.4 (2) **	0.441
Total Fat Mass (%)	41.7 (6)	38.5 (7) **	42.3 (6)	38.2 (7) **	0.191
Android FM (kg)	4.5 (1.3)	3.9 (1.8)	4.8 (1.2)	3.6 (1) **	0.151
**IDF criteria**					
Glucose (mg/dL)	121.5 (33)	115.4 (24) **	123.8 (37)	111.7 (29) **	0.559
TG (mg/dL)	175.3 (90)	139.6 (87) *	194.2(122)	145.4 (83) **	0.863
SBP (mmHg)	150 (17)	137 (13) **	147 (21)	135 (15) **	0.654
DBP (mmHg)	85 (9)	78 (10) *	84 (9)	78 (10) **	0.436
HDL-c (mg/dL)	46.3 (9)	51.5 (11) **	43.3 (9)	46.2 (10)	0.042
LDL-c (mg/dL)	140.4 (36)	140.1(36) **	136.7 (41)	136.3 (41) *	0.570
TC (mg/dL)	221.8 (39)	226.5 (40)	218.5 (47)	213.3 (39)	0.332
Homa-index	4.6 (3.7)	2.6 (2.9) **	4.4 (3.0)	2.3 (1.7) **	0.820
**Cardiometabolic risk factors**					
TyG index	9.1 (0.5)	8.8 (0.5) *	9.1 (0.7)	8.8 (0.6) **	0.487
Waist to height ratio	0.6 (0.0)	0.6 (0.1) **	0.6 (0.1)	0.6 (0.1) **	0.095
LDL-c/HDL-c	3.0 (1.0)	3.5 (0.7)	3.1(0.9)	3.7 (0.8)	0.432
Diabetes %	74.4	65.7	74.4	52.7 *	0.389
Hypertension %	93	43	82.9	52 *	0.323
**Macronutrient intake**					
Total Energy (kcal)	2102 (450)	1529 (316) **	2276 (565)	1548 (381) **	0.527
Carbohydrate (g)	186.5 (58)	140.4 (45) **	201.3 (65)	138.2 (37) **	0.098
Proteins (g)	93.7 (21)	66.6 (18) **	95.7 (20)	78.2 (17) **	0.198
Lipids (g)	97.1 (27)	69.4(17) **	101.3 (29)	66.5 (20) **	0.231
**Fiber consumption (g/1000 kcal)**					
Total fiber	18.7 (10)	20.7 (8) *	21.8 (7)	26.0 (7) *	0.585
Soluble fiber	2.1 (0.9)	2.1 (0.8)	2.0 (0.6)	2.5 (0.9)	0.657
Insoluble fiber	4.3 (3.2)	4.5 (2.1)	3.2 (1)	5.6 (1.5) *	0.190

(Mean ± SD); Paired and Unpaired *t*-tests were carried out. * *p <* 0.05, comparison within each dietary group (baseline and after 6 months); ** *p <* 0.001, within each dietary group (baseline and after 6 months). Δ*p*-value, comparison of the changes (baseline and 6 months) between dietary groups (AHA vs. RESMENA); BMI: body mass index; FM: fat mass; SBP: Systolic blood pressure; DPB: Diastolic blood pressure; HDL-c: high-density lipoprotein cholesterol; IDF, International Diabetes Federation; LDL-c: low-density lipoprotein cholesterol; TC: total cholesterol; TG: triglycerides; WC: waist circumference; TyG: Triglycerides/glucose ratio.

**Table 2:** Non-invasive markers of liver damage at baseline and after 6 months according to dietary treatment.

*n* = 70	AHA (*n* = 33)		RESMENA (*n* = 37)		Δ*p*-Value
	Baseline	6 Months	Baseline	6 Months	
**Hepatic measurements**					
ALT (U/L)	37.4 (21)	25.2 (8) *	29.4 (11)	22.8 (8) **	0.642 ^#^
AST (U/L)	25.1 (10)	23.3 (6)	21.7 (6)	20.2 (4) **	0.126 ^#^
GGT (U/L)	40.2 (24)	30.3 (17) *	41.4 (26)	27.1 (14) **	0.135
M65 (U/L)	307.8 (198)	217.6 (108) **	259.4 (135)	230.9 (91)	0.125
M30 (U/L)	200.3 (125)	128.7 (51) *	156.1 (99)	103.5 (35) *	0.304
NAFLD_LFS	2.2 (2.6)	0.4 (2.2) **	1.9 (1.9)	−0.09 (1.9) **	0.429
FLI	84.6 (17)	68.4 (25) **	85.2 (16)	69.4 (25) **	0.793
HSI	49.2 (6)	44.1 (5) **	48.4 (5)	43.1 (5) **	0.759
VAI	2.8 (1.9)	2.0 (1.7) **	3.6 (2.9)	2.3 (15) **	0.920
BARD	2.5 (0.9)	2.8 (1.1)	2.6 (1.2)	2.8 (1)	0.713
BAAT	2.1 (0.7)	1.6 (0.7) **	2.0 (0.6)	1.7 (0.7) **	0.265

(Mean ± SD); M30 and M65: cytokeratin-18 (CK18) fragments; ALT: alanine aminotransferase; AST: aspartate aminotransferase; GGT: gamma-glutamyl transferase; FLI: fatty liver index; HSI: hepatic steatosis index; VAI: visceral adipose index; NAFLD_LFS: non-alcoholic fatty liver disease liver fat score; BARD score; BAAT score; Paired and unpaired *t*-tests were carried out. * *p <* 0.05, comparison within each dietary group baseline vs. after 6 months. ** *p <* 0.001, within each dietary group baseline vs. after 6 months; Δ*p*-value, comparison changes (baseline and 6 months) between dietary groups. ^#^ Adjusted by baseline value.

**Table 3:** Non-invasive markers of liver damage categorized according to the median of fiber consumption after 6 months.

*n* = 70	Total Fiber (g/Day)		Insoluble Fiber (g/Day)		Soluble Fiber (g/Day)		Fruit Fiber (g/Day)	
	<39.1	≥39.1	<7.5	≥7.5	<3.2	≥3.2	<8.8	≥8.8
**ALT (U/L)**	26.3 (9)	23.3 (8)	26.2 (9)	23.4 (8)	26.4 (9)	23.5 (7)	27.2 (8)	22.4 (8) ^#^
**AST (U/L)**	21.5 (6)	21. 6(6)	22.8 (5)	21.6 (6)	22.6 (5)	21.4 (6)	23.8 (5)	19.3 (5) ^#^
**GGT (U/L)**	29.4 (17)	28.2 (15)	28.1 (14)	29.5 (18)	28.1 (16)	28.2 (17)	34.1 (18)	23.5 (11) *
**M65 (U/L)**	218.6 (83)	232.1 (118)	223.1 (14)	227.4 (20)	231.4 (15)	220.6 (20)	236.5 (22)	213.4 (12)
**M30 (U/L)**	120.2 (50)	109.4 (45)	123.2 (9)	107.5 (7)	124.3 (9)	107.4 (6)	121.2 (8)	113.6 (7)
**FLI**	75.3 (17)	57.2 (29)	74. 4 (21)	60.6 (27) *	71.1 (25)	62.2 (25)	72.4 (23)	65.8 (26)
**HSI**	45.2 (5)	42.4 (6)	4.27 (3)	41.2 (4) *	44.6 (5)	42.7 (6)	43.6 (6)	44.1 (5)
**VAI**	0.5 (2)	−0.1 (1)	2.4 (1)	1.7 (1)	2.1 (1.8)	1.9 (1.4)	2.4 (2.1)	1.9 (1.1)
**NAFLD_LFS**	2.6 (1)	2.8 (1)	0.9 (2)	−0.5 (1) *	0.2 (2.4)	0.1 (1.7)	0.8 (2.2)	−0.3 (1.7)

* *p* < 0.05 indicates differences between values above and below the median. ^#^ *p* < 0.05. Adjusted by baseline values; Cut off mean values: Total fiber (41.0 g); Insoluble fiber (3.1 g); Soluble fiber (1.6 g); Fruit fiber (3.4 g). ALT: alanine aminotransferase; AST: aspartate aminotransferase; GGT: gamma-glutamyl transferase; FLI: fatty liver index; HSI: hepatic steatosis index; VAI: visceral adipose index; NAFLD_LFS: non-alcoholic fatty liver disease liver fat score.

**Table 4:** Regression analysis with changes in fatty liver index and changes in the consumption of fiber from fruits at 6 months.

Δ Fatty Liver Index	β	*p*	P_model_	*R*-Adjusted
Δ Fiber Fruits	0.5769	0.025	0.0265	0.1168
Age	−0.4860	0.03	-	-
Δ Total energy (kcal)	−0.0691	0.149	-	-
Δ MET	0.0003	0.461	-	-

MET, metabolic equivalent of the task.
